# Ubiquitin Accumulation on Disease Associated Protein Aggregates Is Correlated with Nuclear Ubiquitin Depletion, Histone De-Ubiquitination and Impaired DNA Damage Response

**DOI:** 10.1371/journal.pone.0169054

**Published:** 2017-01-04

**Authors:** Adi Ben Yehuda, Marwa Risheq, Ofra Novoplansky, Kirill Bersuker, Ron R. Kopito, Michal Goldberg, Michael Brandeis

**Affiliations:** 1 The Department of Genetics, The Alexander Silberman Institute of Life Sciences, The Hebrew University of Jerusalem Safra Campus, Jerusalem, Israel; 2 Department of Biology, Stanford University, Stanford, California, United States of America; The University of Hong Kong, HONG KONG

## Abstract

Deposition of ubiquitin conjugates on inclusion bodies composed of protein aggregates is a definitive cytopathological hallmark of neurodegenerative diseases. We show that accumulation of ubiquitin on polyQ IB, associated with Huntington’s disease, is correlated with extensive depletion of nuclear ubiquitin and histone de-ubiquitination. Histone ubiquitination plays major roles in chromatin regulation and DNA repair. Accordingly, we observe that cells expressing IB fail to respond to radiomimetic DNA damage, to induce gamma-H2AX phosphorylation and to recruit 53BP1 to damaged foci. Interestingly ubiquitin depletion, histone de-ubiquitination and impaired DNA damage response are not restricted to PolyQ aggregates and are associated with artificial aggregating luciferase mutants. The longevity of brain neurons depends on their capacity to respond to and repair extensive ongoing DNA damage. Impaired DNA damage response, even modest one, could thus lead to premature neuron aging and mortality.

## Introduction

Neurodegeneration is a major challenge facing the global aging population. Millions are afflicted by the highly prevalent Parkinson’s and Alzheimer’s diseases, as well as dozens of less common disorders like Huntington’s disease and eight other polyQ disorders. Protein aggregation is the most prominent histopathological hallmark of most neurodegenerative diseases. Each disease is associated with the aggregation of one or more specific mutant or wild type protein. The effect of these aggregating proteins is, in most cases, a dominant gain of function [1] but it is not clear at all if and how they bring about neuron mortality. It is also debated whether aggregation reduces or increases the toxicity of the protein [2].

In addition to the specific disease associated proteins, several other proteins also localize to the aggregates. The most common of these co-aggregating proteins is ubiquitin, which is present in almost all types of disease-associated aggregates [3]. Inclusion bodies (IB) contain a considerable amount of ubiquitin and several groups have shown that they perturb the ubiquitin homeostasis of the cell [4, 5]. In some experimental systems IB formation is associated with breakdown of the capacity of ubiquitin dependent [6] and independent [7] proteasomal degradation. We have observed [7] that this breakdown is a relatively late event and could therefore be a downstream result of other more direct effects of IB. Cells express considerable amounts of ubiquitin from four different genes. The level of cellular ubiquitin is nevertheless tightly controlled and knockout of Ubb, one of the four ubiquitin genes in mice, led to a 30% reduction of the level of ubiquitin in the hypothalamus resulting in hypothalamic neurodegeneration [8]. This observation demonstrates how sensitive neurons can be to perturbations of their ubiquitin homeostasis.

Ubiquitin plays multiple roles in addition to its function in proteasomal degradation. Cells stained with antibodies to ubiquitin, or expressing fluorescently tagged ubiquitin, have a strong nuclear staining, most of it due to mono-ubiquitinated histones. Histone ubiquitination in response to DNA damage was first discovered by Nico Dantuma [9] and its role in the DNA damage response has been extensively documented [10–16]. This ubiquitination is highly dynamic and is rapidly turned over by multiple ubiquitin ligases and de-ubiquitinating enzymes [17, 18]. Indeed perturbation of ubiquitin homeostasis by proteasomal inhibition leads to rapid depletion of nuclear ubiquitin [19].

At least four histones are monoubiquitinated in the nucleus. Histone H1 is ubiquitinated by RNF8 coupling initiation and amplification of ubiquitin signaling after DNA damage [20]. Histone H2A ubiquitination on K13 and K15 by the RNF168 drives DNA damage signaling [21]. Histone H2B ubiquitination on K120 by the ubiquitin ligases RNF20/40 [22–24] and BAF250 [25] is associated with chromatin activation and is also required for double strand DNA repair [15, 24]. Finally histone H4 is ubiquitinated on K91 by the BBAP ubiquitin ligase [26] leading to H4K20 methylation, which plays a role in the localization of the DDR protein 53BP1 to damaged sites. Whereas histone ubiquitination is associated with repair of double strand breaks, ubiquitination also plays a role in several stages of nucleotide excision repair [9, 27–30].

Brain neurons make up 2% of body mass but consume 20% of its oxygen [31] generating a considerable amount of reactive oxygen radicals. It is conceivable that these radicals are at least partially responsible for the large number of DNA lesions observed in neurons [32, 33]. Given the fact that neurons are not replaceable and designed to last a lifetime, it is understandable that they heavily rely on the DNA damage response (DDR) and repair mechanisms. This can be demonstrated by the fact that several inherited mutations in DDR genes like ATM and NBS have a pronounced effect on brain neurons [34].

We report here that ubiquitin conjugates accumulate on PolyQ IB and that this accumulation is correlated with ubiquitin depletion from the nucleus and extensive histone H2A and H2B de-ubiquitination. Given the important role of histone ubiquitination for the DNA damage response, we tested how these cells respond to DNA damage. We observed that cells with IB indeed fail to respond normally to such damage. The perturbed effect could be partially rescued by ubiquitin overexpression. These observations can explain the observed damage found in DNA of cells with Huntingtin aggregates [35, 36]. Ubiquitin is present in most types of disease related cellular protein aggregates. We thus wondered whether other types of aggregates have a similar effect on nuclear ubiquitin homeostasis and on the DNA damage response. We addressed this question using an “artificial” firefly luciferase mutant protein that is not associated to any disease [37]. Strikingly this protein had the same effects on histone ubiquitination and the DNA damage response as did the Htt-Q91 protein.

## Results

### PolyQ aggregates deplete nuclear ubiquitin and lead to histone de-ubiquitination

A fusion protein of exon 1 of the mutant huntingtin gene, which encodes 91 glutamines and a fluorescent protein (Htt-Q91-FP) is a well-characterized model for PolyQ aggregation [38]. We have recently set up a system to follow this aggregation and its effects on cells in real time. We observed that once the level of this protein achieved a certain threshold concentration, it rapidly aggregates and forms inclusion bodies (IB) [7]. Most aggregates form in the cytoplasm but some aggregates form in the nucleus. We did not observe any difference in behavior of cytosolic and nuclear aggregates. Like most cellular protein aggregates associated with neurodegenerative diseases, also polyQ IB contain ubiquitin [39, 40]. To assess the effect of protein aggregation on the homeostasis of ubiquitin in live cells we generated a U2OS cell line stably expressing ubiquitin with an N-terminal YFP tag (YFP-Ubi). YFP-Ubi gets incorporated into poly ubiquitin chains and is capable of mono-ubiquitination of proteins ([41] and S1 Fig). Live cell studies of these cells show that, as expected, YFP-Ubi is present in all cellular compartments but is considerably enriched in the nucleus and on chromosomes (S1 Mov). We observed a similar enrichment in fixed cells stained with the Fk2 monoclonal antibody that identifies ubiquitinated proteins. Most of the ubiquitination of nuclear proteins comprises of histone mono-ubiquitination [42]. As expected YFP-UbiΔG75,76, a ubiquitin mutant lacking its two C-terminal glycine residues, does not localize to chromosomes (S2 Mov).

**Fig 1:**
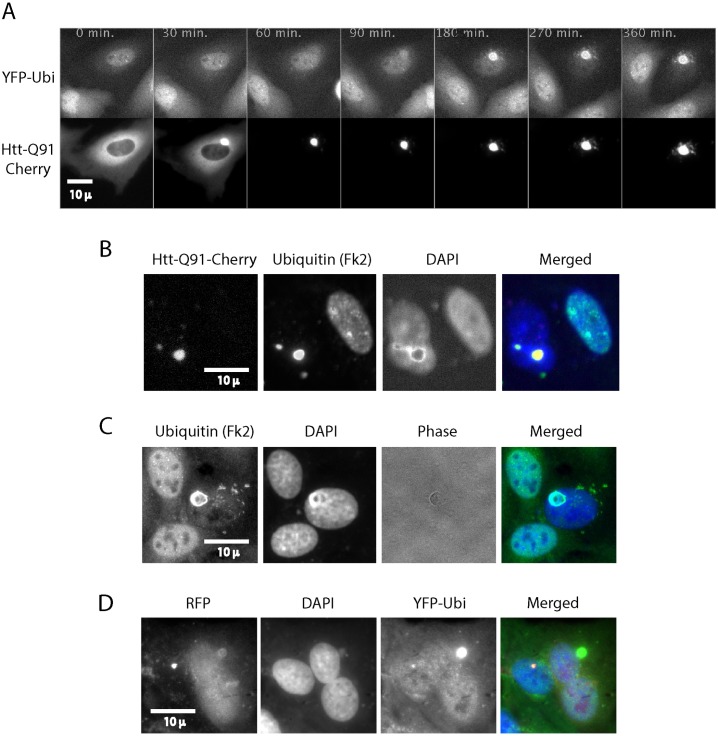
Poly Q aggregates accumulate ubiquitin and lead to depletion of nuclear ubiquitin. Cells stably expressing YFP-Ubi were transiently transfected with Htt-Q91-Cherry and followed by live cell imaging (S3 Mov). Shortly after the Htt-Q91-Cherry aggregates it starts to accumulate ubiquitin. T = 0 was arbitrarily set to the time protein aggregation started in this particular cell. Strikingly YFP-Ubi staining in the nucleus is depleted **(A)**. U2OS Cells were transiently transfected with Htt-Q91-Cherry and fixed for immunofluorescence with an antibody that identifies ubiquitinated proteins (Fk2). The experiment was repeated in N2A and PC12 cells (S2 Fig). In each of these lines the cells with the IB lack nuclear ubiquitin staining compared to the non-expressing cells. **(B)**. U2OS Cells were transiently transfected with untagged Htt-Q91 and fixed for immunofluorescence with Fk2. Ubiquitin accumulated on the untagged IB and was depleted from the nucleus like with the Htt-Q91-Cherry **(C)**. U2OS cells were co-transfected with Ubi[9]-mRFP[1] and YFP-Htt-Q91. Overexpression of ubiquitin reduces depletion of nuclear ubiquitin by Htt-Q91 in spite of the accumulation of ubiquitin on the IB **(D)**.

Transient expression of HttQ91-mCherry in the stable YFP-Ubi cells showed that ubiquitin accumulates on the polyQ aggregates within about an hour of IB formation ([7] and Fig 1A). The YFP-UbiΔG75,76 mutant does not accumulate on aggregates. Moreover inhibition of ubiquitination by an E1 inhibitor [43] also prevented accumulation of ubiquitin on aggregates ([44] and data not shown). These observations indicate that aggregates don’t recruit free ubiquitin but only one conjugated to other proteins.

We observed that accumulation of YFP-Ubi on the aggregate was followed by its depletion from the nucleus (Fig 1A and S3 Mov). We confirmed that IB deplete endogenous ubiquitin from nuclei by transiently transfecting cells with Htt-Q91-Cherry and staining them with the Fk2 antibody (Fig 1B). While YFP-Ubi imaging represents both the conjugated and free ubiquitin, Fk2 stains only ubiquitin conjugated, either as poly or mono ubiquitin, to other proteins. We observed a similar depletion of ubiquitin by the polyQ aggregate in neuronal derived cells lines N2A and PC12 (S2 Fig).

We next expressed untagged Htt-Q91 in cells to ensure that ubiquitin does not accumulate on aggregates and deplete nuclear ubiquitin due to the fluorescent tag of the aggregating protein. Fig 1C shows that untagged polyQ aggregates accumulate ubiquitin and deplete nuclear ubiquitin just like the fluorescently tagged polyQ.

We next explored whether the loss of nuclear ubiquitin is associated with the depletion of the available ubiquitin in the cell. We used the Ub[9]-mRFP[1] expression vector [45], which expresses the open reading frame of a ubiquitin precursor encoding nine ubiquitin monomers in frame with mRFP. Ubiquitin hydrolases convert this fusion protein into nine ubiquitin monomers and one mRFP molecule. The level of mRFP thus indicates how much ubiquitin is expressed in each cell. Fig 1D shows that indeed in cells overexpressing ubiquitin, as indicated by mRFP expression, nuclear ubiquitin staining was maintained in spite of ubiquitin accumulation on IB.

### Accumulation of ubiquitin on IB is correlated with histone de-ubiquitination

Nuclear ubiquitin is mainly conjugated as mono-ubiquitin to chromatin histones [42]. Histone ubiquitination is highly dynamic and is rapidly turned over. In a FRAP (Fluorescence Recovery After Photobleaching) experiment on nuclear YFP-Ubi about 70% of the fluorescence recovered within less than 10 minutes (S3 Fig). It is likely that ubiquitin turnover on different histones and other nuclear proteins follows different kinetics, however most of it seems to be rather rapid. Perturbation of ubiquitin homeostasis by treating cells with the proteasome inhibitor MG132 leads to rapid loss of histone ubiquitination [19] and S4 Mov.

**Fig 2:**
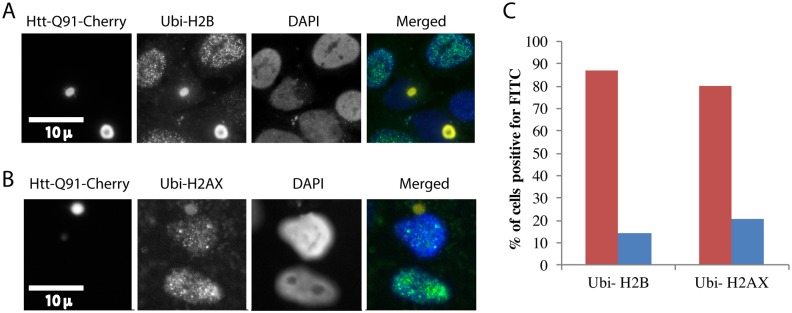
Poly Q aggregates lead to depletion of histone ubiquitination. U2OS cells were transiently transfected with Htt-Q91-Cherry and fixed for immunofluorescence with an antibody that identifies ubiquitinated H2B **(A)** and ubiquitinated H2AX The staining of the IB with the Ubi-H2B and Ubi-H2AX antibodies is likely due to cross reaction of these antibodies with the high concentrations of ubiquitin on the aggregates **(B)**. Cells were either imaged under the microscope **(A,B)** or analyzed in bulk by flow cytometry. The red bars indicate cells that lack aggregates and the blue bars cells with aggregates. The presented experiment is a representative of three repeats **(C)**.

We used antibodies against specific ubiquitinated histones to examine whether PolyQ IB lead to histone de-ubiquitination. Fig 2A shows cells expressing PolyQ IB stained with antibodies against ubiquitinated histone H2B. Cells with IB have a significantly reduced staining of histone H2B ubiquitination. Fig 2B shows that also histone H2AX ubiquitination is considerably reduced in cells with IB.

**Fig 3:**
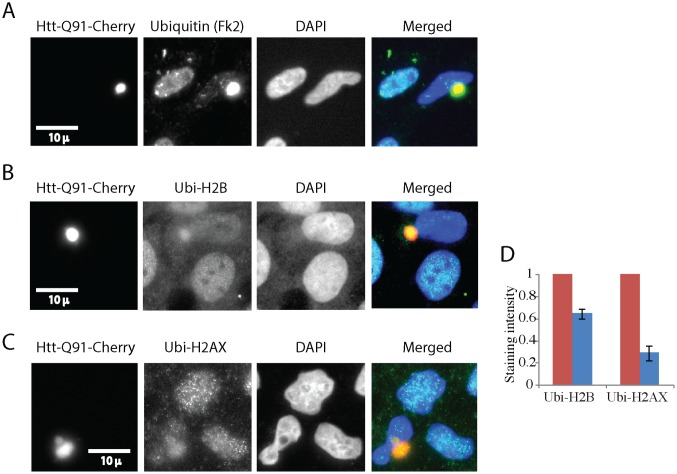
Inducible expression of Poly Q aggregates leads to depletion of histone ubiquitination. U2OS cells expressing an inducible Htt-Q91-Cherry vector were induced with dox. Cells were fixed and stained with Fk2 antibodies that identify ubiquitinated proteins B, antibodies against ubiquitinated H2B **(B)** and ubiquitinated H2AX **(C)**. Cells were imaged under the microscope **(B,C)**. Several hundred cells in multiple fields were photographed and quantified by ImageJ. The red bars indicate cells that lack aggregates (normalized to 1) and the blue bars cells with aggregates, error bars are SE **(D)**.

The experiments described so far were performed by transient expression of Htt-Q91 in cells. This method leads to rapid expression of high levels of the aggregating protein. It could be argued that this acute expression is of limited physiological relevance. We therefore sought a more physiological method of expression. We generated cells expressing Htt-Q91-Cherry from a tetracyclin inducible promoter, which is induced by doxycycline. While all cells expressed the protein at detectable levels only a fraction of them expressed sufficient amounts to form aggregates. These cells thus express Htt-Q91-Cherry close to the threshold level required for IB formation. At the phenomenological level this is more reminiscent to what is observed in the brain, where only a small number of cells are observed to contain IB. It is however of course unknown what the real levels of Htt in the brain are and at what concentration they form IB. Unlike the transiently expressing cells IB formation in these inducible lines does not lead to rapid cell death. We induced these cells for several days, fixed them and stained them with antibodies against Ubiquitin (Fk2), Ubi-H2B and Ubi-H2AX. Fig 3 shows that cells with IB have considerably reduced levels of nuclear ubiquitin and of Ubi-H2B and Ubi-H2AX, compared to neighboring cells that lack IB.

### IB formation is correlated with a compromised DNA damage response

**Fig 4:**
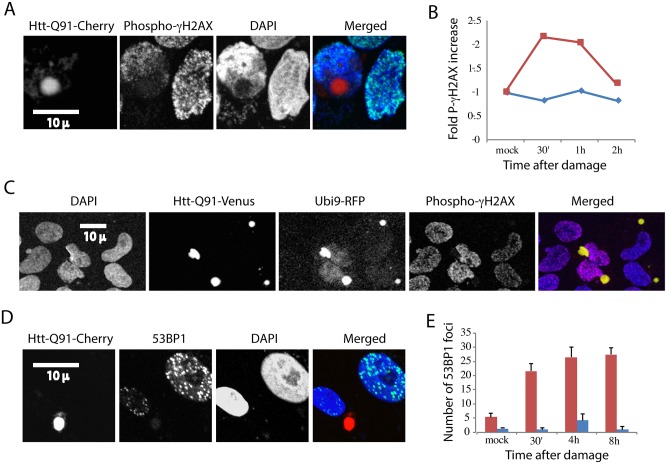
Cells with Poly Q aggregates have an impaired DNA damage response, which can be partially rescued by ubiquitin over-expression. U2OS cells were transiently transfected with Htt-Q91-Cherry, treated with DNA damaging agent neocarzinostatin and fixed at indicated time points. Cells were stained with an antibody against the early marker for DDR phosphor-gamma-H2AX. The red line indicates cells that lack aggregates and the blue line cells with aggregates. The presented experiment is a representative of three repeats **(A, B).** U2OS cells were co-transfected with Ubi[9]-mRFP[1] (red) and Htt-Q91-Venus treated with neocarzinostatin and fixed after one hour **(C)**. Cells transiently transfected with Htt-Q91-Cherry, and treated with neocarzinostatin were fixed at indicated time points and stained with antibodies against the late DDR marker 53BP1 **(D, E)**. Cells were either imaged by microscope **(A, C, D, E)** or analyzed by flow cytometry. The red bar indicates cells that lack aggregates and the blue bar cells with aggregates. Number of foci of 53BP1 were counted manually, error bars represent SE, three repeats **(E)**.

Histone ubiquitination plays a crucial role in various stages of the DNA damage response. We therefore wondered whether the significant reduction in histone ubiquitination caused by IB would affect the capacity of cells to respond to DNA damage. Histone ubiquitination has been mainly implied in response to double strand breaks and we therefore focused on response to this type of damage. We treated cells transiently expressing PolyQ IB with the radiomimetic agent neocarzinostatin and stained cells with antibodies against phospho-gamma-H2AX. Phosphorylation of gamma-H2AX is considered to be an early response to damage. While it is still under debate whether the initiation of it requires histone ubiquitination, the amplification of this signal depends on this modification [46, 47]. Fig 4A compares cells with IB to their untransfected neighbors 30 minutes after neocarzinostatin treatment. It is evident that the cells with IB have a considerably smaller number of phospho-gamma-H2AX positive foci. We used flow cytometry to quantify the effect of DNA damage in response to DNA damage. Fig 4B shows that cells expressing high levels of Htt-Q91-Cherry, representing cells with IB, failed to induce gamma-H2AX phosphorylation in response to damage. Cells without Htt-Q91-Cherry did, as expected, induce gamma-H2AX phosphorylation in response to neocarzinostatin treatment.

We next tried to rescue gamma-H2AX phosphorylation by over expression of ubiquitin. We transfected Ub[9]-mRFP[1] [45] simultaneously with Htt-Q91-YFP. Fig 4C shows that in cells with IB over-expressing intermediate levels of ubiquitin, gamma-H2AX phosphorylation markedly increased (Fig 4C- two cells in the middle) compared to cells with IB over-expressing low levels of ubiquitin (Fig 4C -two right cells) or cells with IB not over-expressing ubiquitin (Fig 4A). Higher levels of ubiquitin expression proved to be toxic to cells. This observation cautiously indicates that depletion of histone ubiquitination could be directly or indirectly involved in reducing gamma-H2AX phosphorylation in cells expressing IBs.

The requirement of histone ubiquitination for the recruitment of the 53BP1 repair protein to DNA double strand breaks has been well documented [48]. We transfected cells with Htt-Q91-Cherry, treated them with neocarzinostatin, fixed them and stained them with anti 53BP1. Fig 4D and 4E show that cells with IB have much less 53BP1 positive foci than cells without aggregates. To test the potential of ubiquitin over-expression on restoring the formation of 53BP1foci we again used Ub[9]-mRFP[1]. However this time our results remained inconclusive. Phosphorylation of gamma-H2AX and 53BP1 foci require different types of histone ubiquitination—H2AX Ser139 [46, 47] and H2A Lys15 [48] respectively. It is thus conceivable that crude over expression of ubiquitin will not necessarily rescue both effects to the same extent.

### Nuclear ubiquitin depletion, histone de-ubiquitination and compromised DNA damage response are associated with “generic” firefly luciferase aggregates

**Fig 5:**
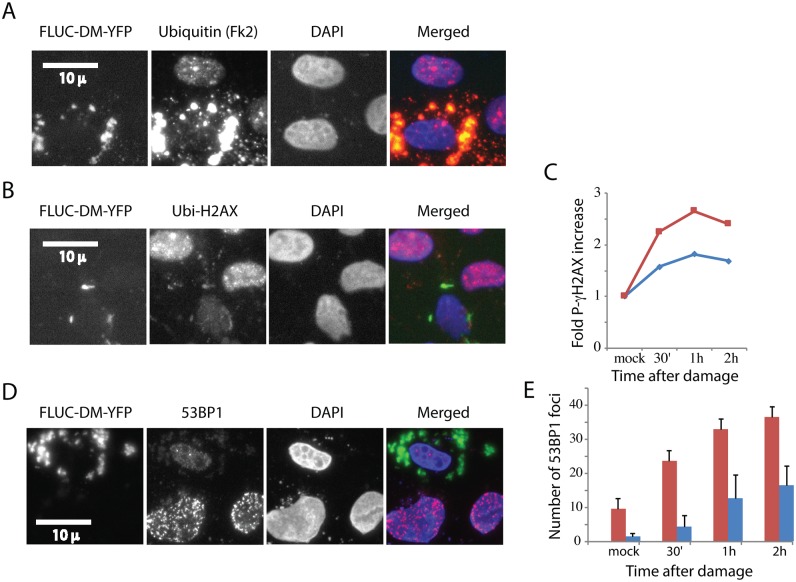
Firefly luciferase aggregates are associated with histone de-ubiquitination and a compromised DNA damage response. U2OS Cells were transiently transfected with FLUC-DM-YFP and fixed for immunofluorescence with an Fk2, an antibody that identifies ubiquitinated proteins **(A)** and ubiquitinated H2AX **(B)**. U2OS cells were transiently transfected with FLUC-DM-YFP, treated with the DNA damaging agent neocarzinostatin and fixed at indicated time points **(C,E)**. Cells were either stained with an antibody against phosphor-gamma-H2AX and analyzed by flow cytometry **(C)** The presented experiment is a representative of three repeats. Alternatively, cells were stained or against the 53BP1**(D)**, Cells from three experiments were counted and quantified **(E)**. Error bars represent SE, three repeats.

Most disease associated protein aggregates are rich in ubiquitin. We therefore wondered whether the effect we observed of PolyQ IB on nuclear ubiquitin homeostasis and the DNA damage response are associated with other types of aggregates. We sought an aggregating protein that is not normally expressed in human cells and which is not associated with any disease. We chose a mutant of firefly luciferase fused to GFP that was designed to form aggregates in cells [37]. Unlike PolyQ, which forms a clean and single IB, FLUC forms multiple aggregates in each expressing cell. Fig 5A shows that FLUC aggregates accumulate ubiquitin like PolyQ IB and that consequently ubiquitin is depleted from nuclei. Fig 5B shows that, as expected, these aggregates reduce histone H2AX ubiquitination. We next checked the effect of FLUC on the DNA damage response. Fig 5C shows that cells expressing FLUC fail to respond to such damage by phosphorylating histone gamma-H2AX. Similarly, FLUC drastically reduced the number of 53BP1 foci in cells that suffered DNA damage (Fig 5D and 5E). These results indicate that FLUC aggregates and polyQ IB have a comparable effect on histone ubiquitination and the DNA damage response. These observations further imply that many other disease- associated aggregating proteins could have a related effect on nuclear ubiquitin and the capacity of cells to respond to damage.

### PolyQ aggregates sequester 53BP1

**Fig 6:**
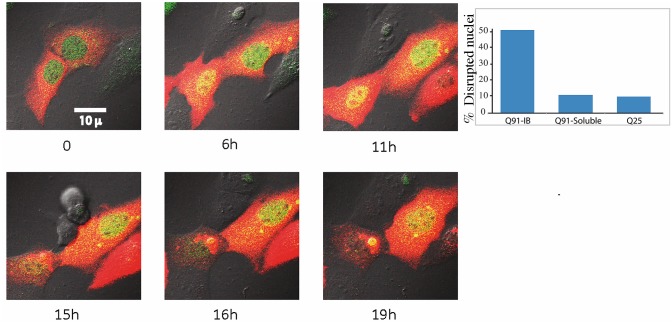
53BP1 exits the nucleus and accumulates on polyQ aggregates. Cells stably expressing 53BP1-GFP (green) were transiently transfected with Htt-Q91-Cherry (red) and followed by live cell imaging for aggregate formation. The 53BP1-GFP exits from the nucleus and localizes to the aggregate shortly after it forms (S5 Mov) **(left panels)**. Cells stably expressing YFP-NLS were transfected with Htt-Q91-Cherry or Htt-Q25-Cherry and fixed after 24 hours. The number of cells with IB, soluble Q91 and Q25 with disrupted nuclei were counted **(right panel)**. See also S6 Mov).

Staining cells expressing polyQ IB with anti 53BP1 not only showed considerably reduced levels of damage specific foci, but also often showed staining of the IB itself (Fig 4C). To test whether this is a nonspecific interaction between the antibody and aggregated polyQ we stably expressed 53BP1-GFP in cells and followed them by live cell imaging. Fig 6 (left) and S5 Mov show that when polyQ transiently expressed in these cells aggregated, 53BP1-GFP accumulated on it. This accumulation is not only interesting due to the yet inexplicable fact that 53BP1 is attracted to aggregates, but also due to the inevitable conclusion that 53BP1 exits the nucleus. The prompt translocation of 53BP1 from the nucleus to the aggregates in the cytoplasm is unlikely due to active regulated export, and suggests the disruption of the nuclear compartment. To test whether the nuclear compartment is disrupted, we generated cells stably expressing YFP with a nuclear localization signal (YFP-NLS). These cells were transfected with Htt-Q91-mCherry and YFP-NLS was followed in parallel to IB formation. S6 Mov shows that formation of a polyQ aggregate coincides with massive leakage of YFP from the nucleus into the cytoplasm. Fig 6 (right) shows that about 50% of cells with aggregates have a disrupted nuclear compartment while only about 10% of cells with diffuse Q91, as well as Q25 demonstrate such a disruption. These observations support a recent report [49] showing that perinuclear inclusions disrupt the nuclear membrane.

## Discussion

Brain neurons are not replaced during the lifetime and can thus reach a ripe old age. Moreover their number does not drastically decline during normal aging [50]. The symptoms of neurodegeneration in contrast are caused by premature loss of neurons in specific parts of the central nervous system. Protein aggregation is the most obvious histopathological hallmark of neurodegeneration but how aggregation is related to neuronal demise is not fully understood. Toxicity has been attributed both to the pre-aggregated [51, 52] as well as the aggregated protein [7]. These observations do not necessarily contradict each other. Even if the pre-aggregated proteins are more deleterious to the cell in the short run, the aggregates could still be toxic in the long run. Our results suggest that the aggregate might have a long-term chronic effect on the capacity of cells to repair their damaged genome. This model does not exclude any other toxic effects of the aggregates and the pre-aggregate protein.

Most neurodegenerative diseases are characterized by their gradual and late onset. In this sense they can be viewed as premature aging of specific cell populations. Accumulation of DNA damage is a normal effect of aging as are breakdown of proteostasis and disruption of the nuclear lamina [53, 54]. Protein aggregation indeed leads to breakdown of proteostasis [6, 7], as well as reduced response to DNA damage [55] and to disruption of the nuclear lamina [49]. All these effects accelerate aging by pushing the cells faster over the cliff.

Here we show that perturbation of ubiquitin homeostasis is likely to be involved in the recently reported compromise of the DNA damage response [36]. We have further shown that the effect of protein aggregation might have similar characteristics no matter which protein forms the aggregates. It remains therefore a mystery why different cell types are affected in different aggregation diseases.

In addition to ubiquitin, IB bind and deplete other proteins, which could affect the DNA damage response like VCP/p97 [36] and 53BP1 as we have shown here.

It is yet unknown why ubiquitin conjugates accumulate on aggregates. Several ubiquitin binding proteins like NPL4 which complexes with VCP/p97, Atx3 and the p62/SQSTM1 are all known to localize to aggregates and could also contribute to the accumulation of ubiquitin. It is possible that this accumulation takes the form of a chain reaction where ubiquitin binding proteins start to settle on the aggregate binding ubiquitinated chains, which further bind ubiquitin binding proteins and so on and so forth. The initial trigger could be a small amount of ubiquitin on the primary aggregating protein or of co-aggregating proteins. We have recently shown that ubiquitin binds tightly to IB so that once it has been bound it remains there [44].

If indeed perturbation of ubiquitin homeostasis accelerates cell aging, preventing this perturbation could be a promising new medical approach. We have recently shown that ubiquitin is recruited to the aggregates after they form [7]. This observation has been corroborated in the R6/2 PolyQ mouse model [56]. As ubiquitin accumulation happens after aggregation and is not part of the aggregation process it should be in theory possible to uncouple it from aggregation. This is of importance in light of the reports that show that pre-aggregated proteins are highly toxic and that aggregation could detoxify them [51]. Inhibition could be achieved either by small molecule inhibitors or by inhibition of proteins that are binding ubiquitin to aggregates. Such inhibition will enable us to discriminate between the effect of the aggregate itself and to the effect of depletion of cellular ubiquitin.

## Materials and Methods

### Plasmids

pEF-EYFP-Ubi was generated by cloning ubiquitin downstream of EYFP in the pEF-BOS vector. Htt-Q91-Cherry was generated by cloning the first exon of huntingtin with 91 glutamine repeats from N-htt(Q91)–chFP [7] into pcDNA4/TO with KpnI+XbaI. Inducible Htt-Q91-Cherry was generated by cloning first exon of huntingtin with 91 glutamine repeats from N-htt(Q91)–chF into pTRE-Tight with KpnI+XbaI. Htt-Q91 was generated by deleting the open reading frame of Cherry from Htt-Q91-Cherry. The Ub[9]-mRFP[1] expression vector [45] was obtained from the Addgene repository. FLUC-DM-YFP was a kind gift of F. Ulrich Hartl and Rajat Gupta of the Max Planck Institute in Martisried, Germany.

### Cell culture and transfections

Human dox inducible U2OS-human osteosarcoma cells were obtained from invitrogene, mouse Neuro 2A (N2A) and rat Pheochromocytoma cells were a kind gift or Sagiv Shifman from our institute. U2OS and N2A cells were cultured in DMEM with 10% FCS and Penicillin- Streptomycin (all from Biological Industries). Rat PC12 cells were cultured in DMEM with 5% FCS, 10% Horse serum and Pen/Strep. U2OS cells were transfected with Jetprime^™^ (Polyplus Tranfection) and N2A and PC12 with Lipofectamine 2000 (Life Technologies). U2OS cells stably expressing inducible pTET-Q91-mCherry were induced with 1μg/ml Doxycycline (Sigma) for 48 hours or more. DNA damage was induced with 0.03μg/ml Neocarzinostatin (Sigma) for 10 minutes in darkness. Cells were subsequently washed and fed with fresh medium.

### Antibodies and indirect immunofluorescence

The following antibodies were used: Mouse anti Mono and poly Ubiquitinylated conjugates FK2 (ENZO), Mouse anti Phospho histone H2A.X (Ser139) (Millipore), Mouse anti Ubiquityl Histone H2B (Lys120) (Millipore), Mouse anti Ubiquityl Histone H2A.X (Lys119) (Millipore), Rabbit anti 53BP1 (Bethyl), Rabbit anti histone H3 (Sigma). Fluorescently tagged secondary antibodies were from Jackson ImmunoResearch.

For indirect immunostaining U2OS and N2A cells were cultured on uncoated coverslips and PC12 cells were cultured on coverslips coated with Poly-Lysine (Sigma). After transfections and the relevant treatment cells were washed briefly with PBS and subsequently for 30 seconds with cytoskeleton buffer (CKS) (100 mM NaCl, 300 mM Sucrose, 10 mM Pipes, 3 mM MgCl_2_, 1 mM EGTA). U2OS cells were fixed for 10 minutes with 4% Paraformaldehyde (pFA) for experiments entailing DNA damage or with 4% Formaldehyde otherwise, and washed three times with PBS. N2A and PC12 cells were fixed for 30 minutes with 4% pFA and were subsequently treated for 30 minutes with 0.5% Triton in PBS to perforate their membranes. Staining with antibodies against ubiquityl histone H2B required a more drastic protocol to denature the chromatin. Cells were therefore fixed for 20 minutes with 4% pFA, membrane perforation with 0.1% triton in PBS for 20 minutes and chromatin denaturation with 2M HCl for 10 minutes at 37°C. This treatment precludes chromatin staining with DAPI and cells were therefore double stained with anti-Histone H3.

All cells were treated for 30 minutes with blocking buffer (1% Triton, 10% FCS in PBS). Cells were incubated with the primary antibody diluted in blocking buffer (contact authors for specific dilutions) overnight (phosphor histone H2A.X, and 53BP1) or for one hours (all other antibodies). Cells were washed three times with PBS for 5 minutes each and incubated for one hour with the relevant secondary antibody (1:1000) and washed again three times with PBS. Cells were stained with DAPI or Hoechst (Sigma) and mounted with fluorescent mounting medium (DakoCytomation).

Stained cells were imaged with an inverted IX70 Olympus microscope (40X/1.3 oil immersion objective) fitted with Sutter filter wheels, a Roper HQ cooled CCD camera driven by Micromanager software. Alternatively, cells were imaged on an FV1000 Olympus confocal laser scanning microscope (60X/1.4 oil immersion objective). Images were processed with ImageJ (contrast enhancement, cropping and color merging only).

### FACS analysis

DNA damage was induced with 0.03μg/ml Neocarzinostatin (Sigma) for 10 minutes in darkness in 24 well tissue culture dishes. Cells were detached from the wells with trypsin, pelleted (1200 RCF 3 minutes at 4°C) and suspended in 4% pFA for 30 minutes on ice. Cells were pelleted again, suspended in FACS buffer (PBS, 1% FCS, 0.05% Sodium Azid) and kept over night at 4°C. The following day cells were pelleted, washed twice in Saponin solution (0.1% saponin in FACS buffer) to perforate the membrane and suspended in 50 μl saponin solution with mouse anti Histone H2A.X (Ser 139) for 30 minutes on ice. Staining was terminated by the addition of 1 ml saponin solution followed by three washes as described above. Cells were next incubated with 50 μl saponin solution with Goat α Mouse DyLight 488 (Pierce Biotechnology) for 30 minutes on ice and washed again three times. Cells were finally suspended in 300 μl of saponin solution and 10000 cells were read for each sample on a FACS Aria III (BD) with both the green (488 nm) laser to visualize the antibody staining and the red (561 nm) laser to visualize Q91-mCherry.

## Supporting Information

S1 Fig YFP-Ubi gets incorporated into Polyubquitin chains. Protein was extracted from 293 cells untransfected (Un) and transfected with expression vectors for YFP, YFP-Ubi (WT) and YFP-UbiΔG75,76. Protein was resolved by SDS-PAGE and immunoblotted with antibodies against GFP (cross-reacting with YFP) and Ubiquitin. The poly-ubiquitin ladder in the GFP blot of wild type YFP-Ubi indicates that it gets incorporated into poly-ubiquitin chains. As expected the YFP-UbiΔG75,76 mutant does not get incorporated.
(TIF)

S2 Fig Poly Q aggregates accumulate ubiquitin and lead to depletion of nuclear ubiquitin. N2A (top) and PC12 (bottom) cells were transiently transfected with Htt-Q91-Cherry and fixed for immunofluorescence with an antibody that identifies ubiquitinated proteins (Fk2).
(TIF)

S3 Fig Ubiquitin is turned over relatively rapidly on chromosomes. A U2OS cell stably expressing YFP-Ubi was photobleached as indicated with the arrow and followed for 10 minutes. Time lapse frames show that about 70% ofr ubiquitination on histones recovers within about 8 minutes **(A, B)**. The fluorescence of one set of segregating sister chromatids of a dividing NIH3t3 murine fibroblast stably expressing YFP-Ubi was photobleached **(C).** Time lapse frames show how rapidly ubiquitination on histones recovers even under conditions of chromatin condensation.
(TIF)

S1 Mov YFP-Ubi is concentrated in the nucleus and binds to chromosomes. Cells stably expressing YFP-Ubi were imaged every five minutes.
(MOV)

S2 Mov The non-conjugating YFP-UbiΔG is diffuse in the cell and does not bind chromosomes. Cells stably expressing YFP-UbiΔG75,76 were imaged every five minutes on an LSM5 (Zeiss) laser scanning microscope.
(MOV)

S3 Mov Ubiquitin accumulates on aggregates after they form and is depleted from the nucleus. Cells stably expressing YFP-Ubi were transfected with Htt-Q91-Cherry and imaged every six minutes. Left panel is Htt-Q91-Cherry and right panel ubiquitin. T = 0 was arbitrarily chosen at three frames before aggregation of Htt-Q91-Cherry in the lower cell. Initially ubiquitin is absent from the aggregate and it starts to accumulate within about one hour. By the end of the movie the level of ubiquitin in the nucleus is considerably reduced and is comparable to the level of ubiquitin in the cytoplasm.
(AVI)

S4 Mov Perturbation of ubiquitin homeostasis in the cell by a proteasome inhibitor leads to loss of nuclear ubiquitin. Cells stably expressing YFP-Ubi were treated at T = 0 with the proteasome inhibitor MG132 and imaged every five minutes.
(MOV)

S5 Mov 53BP1 exits the nucleus and accumulates on polyQ aggregates. Cells stably expressing 53BP1-GFP (top-green) were transiently transfected with Htt-Q91-Cherry (middle-red) and followed by live cell imaging for aggregate formation. The 53BP1-GFP exits from the nucleus and localizes to the aggregate shortly after it forms.
(AVI)

S6 Mov PolyQ aggregates rupture the nuclear lamina. Cells stably expressing NLS-YFP (green) were transiently transfected with Htt-Q91-Cherry (red) and followed by live cell imaging for aggregate formation. NLS-YFP exits from the nucleus upon aggregate formation.
(AVI)
