# Supplementary figures and images for: Ubiquitin Accumulation on Disease Associated Protein Aggregates Is Correlated with Nuclear Ubiquitin Depletion, Histone De-Ubiquitination and Impaired DNA Damage Response
Source: PLoS One. 2017 Jan 4;12(1):e0169054. doi: 10.1371/journal.pone.0169054 (PMC5215683; doi:10.1371/journal.pone.0169054)

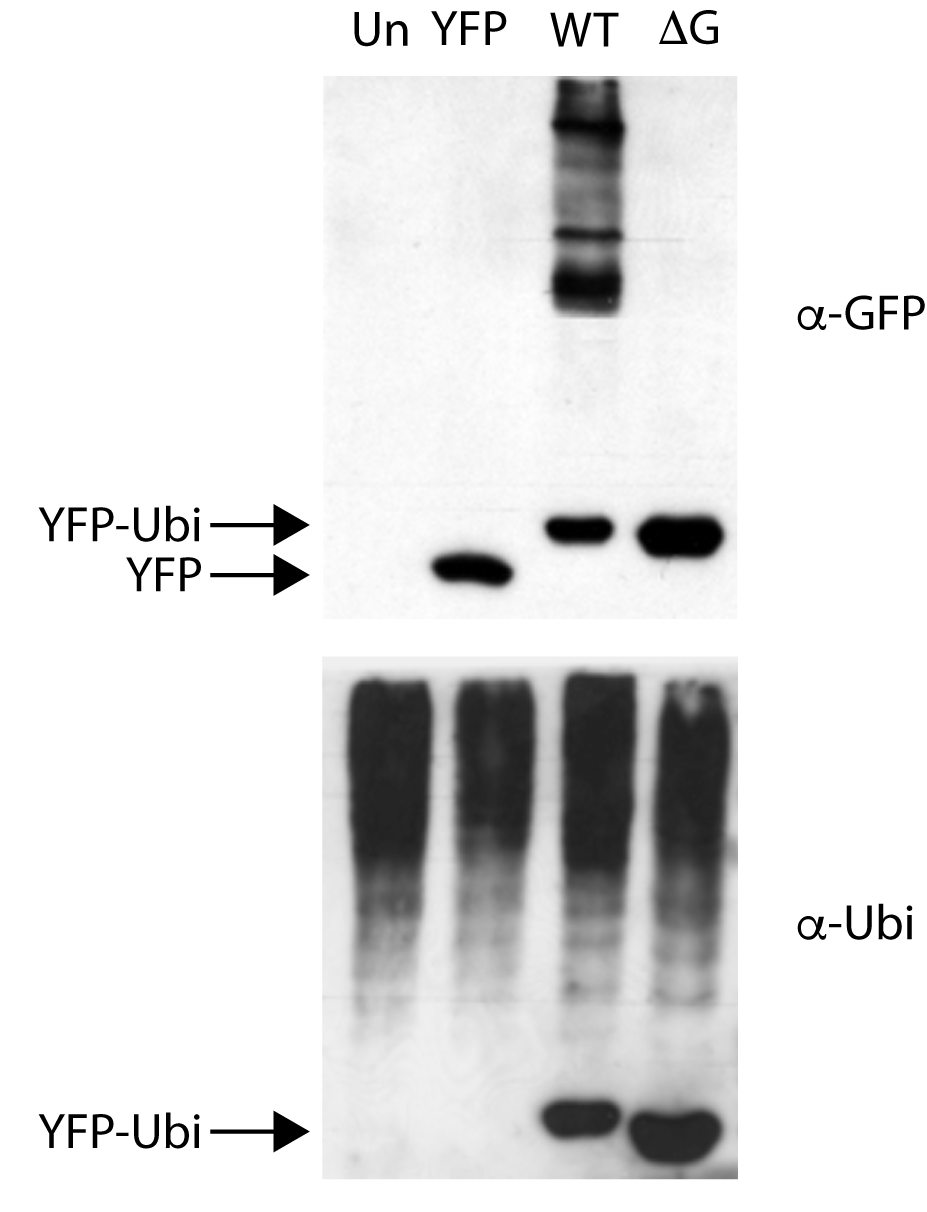

Supplement: S1 Fig — Protein was extracted from 293 cells untransfected (Un) and transfected with expression vectors for YFP, YFP-Ubi (WT) and YFP-UbiΔG75,76. Protein was resolved by SDS-PAGE and immunoblotted with antibodies against GFP (cross-reacting with YFP) and Ubiquitin. The poly-ubiquitin ladder in the GFP blot of wild type YFP-Ubi indicates that it gets incorporated into poly-ubiquitin chains. As expected the YFP-UbiΔG75,76 mutant does not get incorporated. (TIF) [file pone.0169054.s001.tif]

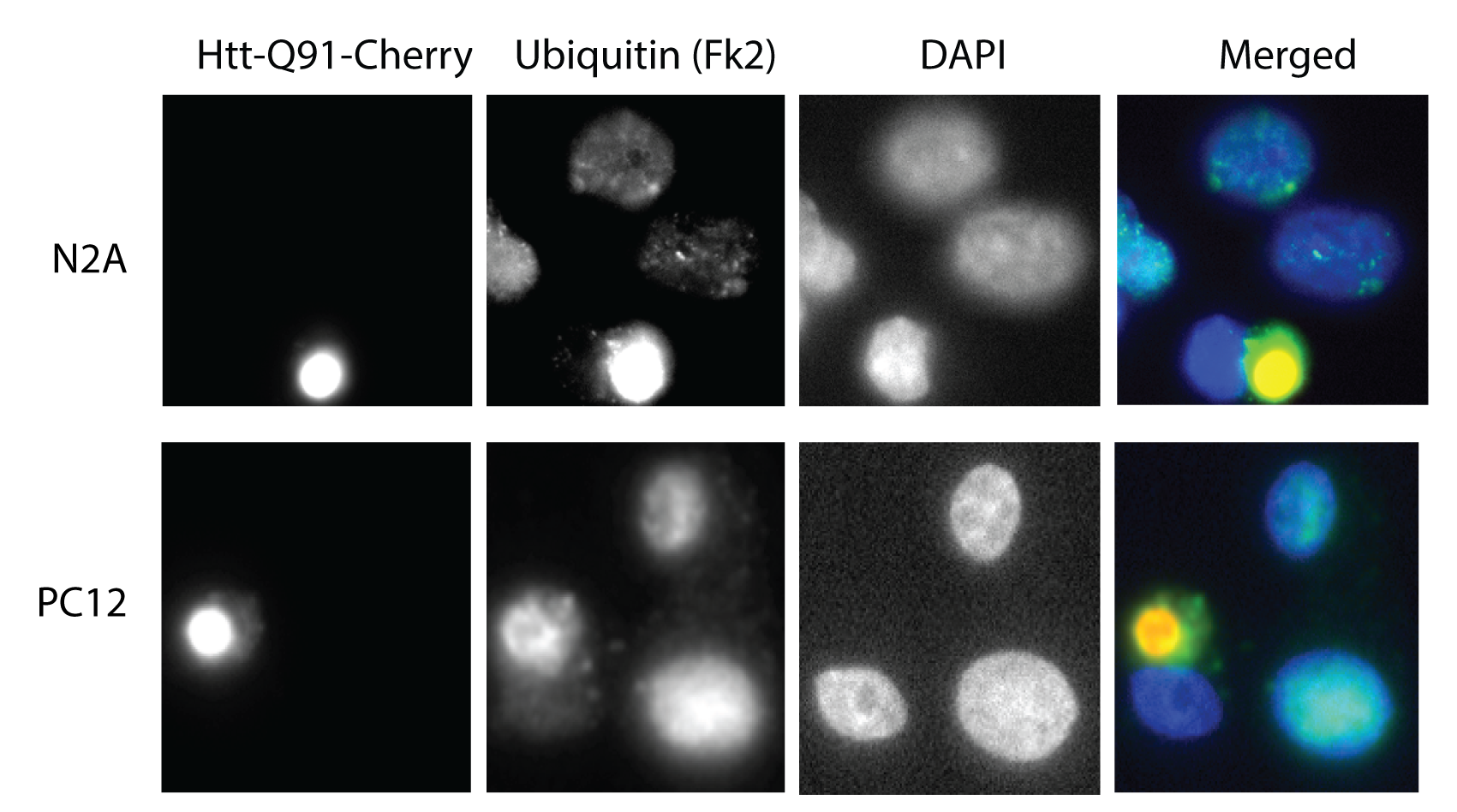

Supplement: S2 Fig — N2A (top) and PC12 (bottom) cells were transiently transfected with Htt-Q91-Cherry and fixed for immunofluorescence with an antibody that identifies ubiquitinated proteins (Fk2). (TIF) [file pone.0169054.s002.tif]

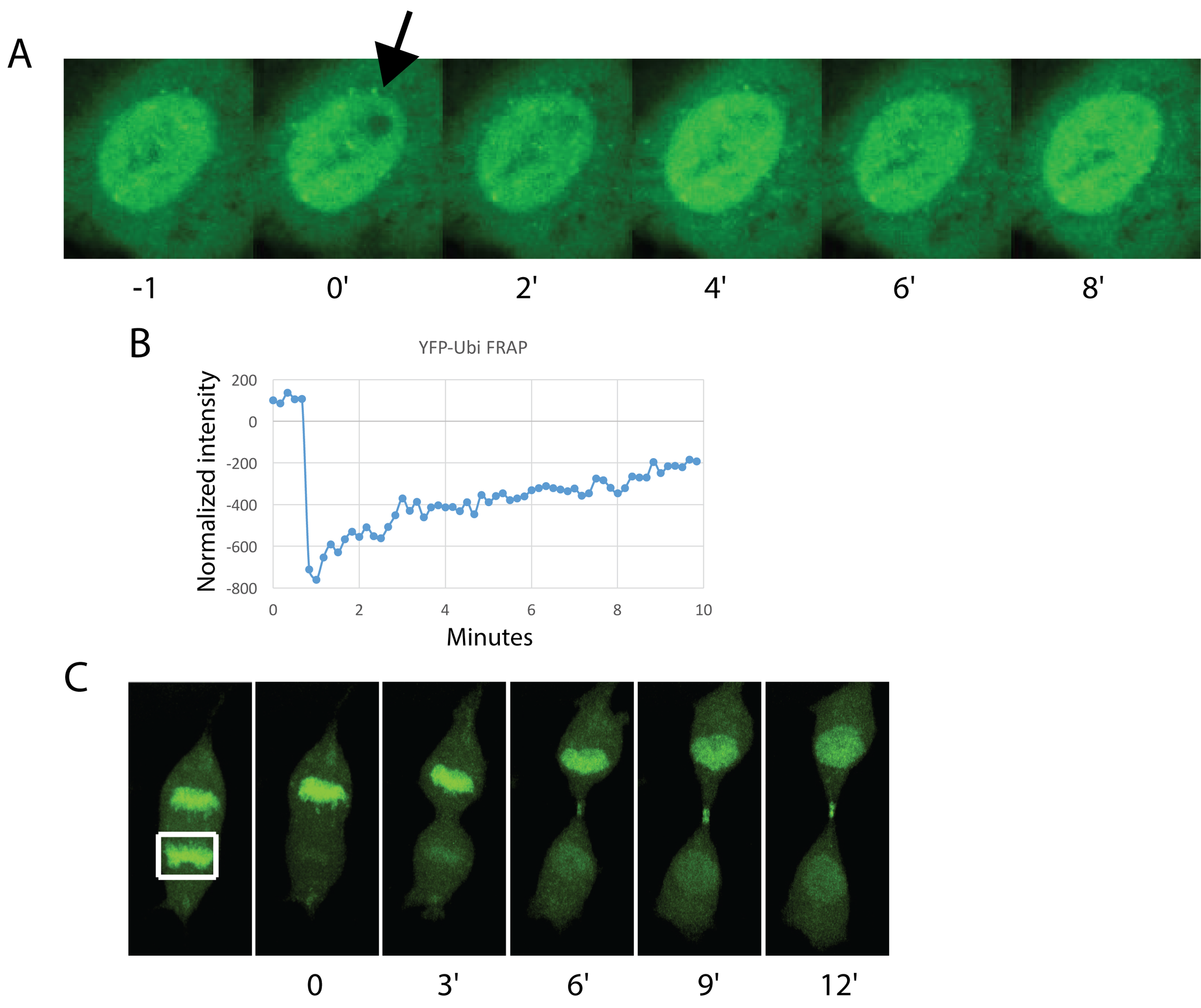

Supplement: S3 Fig — A U2OS cell stably expressing YFP-Ubi was photobleached as indicated with the arrow and followed for 10 minutes. Time lapse frames show that about 70% ofr ubiquitination on histones recovers within about 8 minutes (A, B). The fluorescence of one set of segregating sister chromatids of a dividing NIH3t3 murine fibroblast stably expressing YFP-Ubi was photobleached (C). Time lapse frames show how rapidly ubiquitination on histones recovers even under conditions of chromatin condensation. (TIF) [file pone.0169054.s003.tif]
